# A *Blastomycosis dermatitis* with only skin manifestations: a case report

**DOI:** 10.3389/ffunb.2026.1687338

**Published:** 2026-02-26

**Authors:** Zhuren Ruan, Zhijian Yao, Yuanyuan Chen, Cunwei Cao, Gao Wei, Xianghui Li

**Affiliations:** Department of Dermatology and Venereology, The First Affiliated Hospital of Guangxi Medical University, Nanning, China

**Keywords:** *Blastomyces dermatitidis*, case report, common wart, itraconazole, mNGS

## Abstract

**Background and objective:**

Infections caused by *Blastomyces dermatitidis* can usually affect nearly every organ in the body, including the lungs, skin, bones, and genitourinary system.

**Methods:**

We present a case of a 33-year-old Chinese male who developed *Blastomyces dermatitidis* infection with solely skin involvement one year after returning from the United States.

**Results:**

The patient initially presented with verrucous plaques on his face and occipital region, which were misdiagnosed as common wart. Comprehensive blood tests, including assessments of complete blood count, liver function, kidney function, T lymphocyte subset count, G-test (1,3-β-D-glucan test), GM-test (Galactomannan test), and *Cryptococcus* test (*Cryptococcal* capsular polysaccharide antigen test), all returned normal results. Fungal culture and molecular sequencing confirmed the organism’s identity as *Blastomyces dermatitidis*. The patient was treated with Itraconazole, and the verrucous plaques resolved without recurrence of facial rashes.

**Conclusion:**

This case highlights the importance of considering *Blastomyces dermatitidis* infection in patients with a history of residence in endemic regions and unexplained skin lesions, even without respiratory symptoms or other systemic involvement.

## Introduction

*Blastomyces dermatitidis* is a dimorphic fungal pathogen that grows as mold in the environment and converts to yeast in human tissues at body temperature. Endemic to the Mississippi and Ohio River valleys, it is only rarely reported outside North America, with sporadic cases from Africa and India (Castillo et al., 2016). The typical incubation period for *Blastomyces dermatitidis* infection ranges from 4 to 6 weeks (Pullen et al., 2022). Pulmonary infection is the most common clinical manifestation, accounting for approximately 80% of cases (Linder et al., 2023; Tat et al., 2023). However, the infection can spread to extrapulmonary sites such as the skin, bones, reproductive system, and nervous system via hematogenous dissemination. Given the nonspecific nature of the clinical manifestations, misdiagnosis occurs probably. We describe a 33-year-old Chinese man who developed isolated cutaneous *blastomycosis* one year after returning from the United States. Because the patient lacked pulmonary or systemic symptoms, the lesion was initially dismissed as a common wart, illustrating how primary cutaneous disease can masquerade as a trivial dermatosis.

## Case report

**Figure 1:**
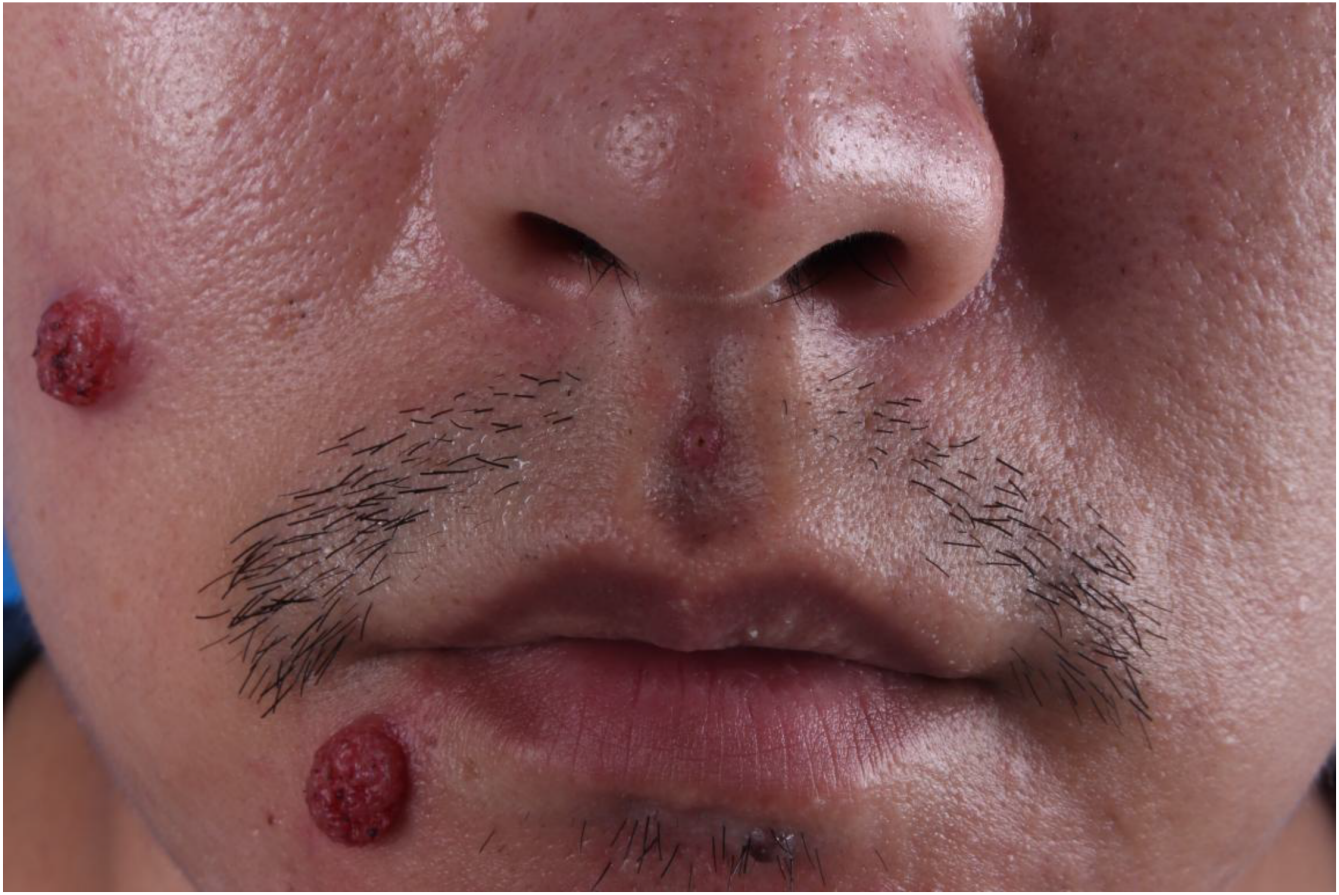
Two skin lesions raised on the right cheek and chin, with uneven surfaces and a color ranging from bright red to dark red, with clear boundaries and does not show a clear trend of merging into patches.

An otherwise healthy 33-year-old man presented with a 2-week history of verrucous plaques on the right face. The initial lesion was a 0.5-cm, firm, hyperkeratotic papule on the right nasolabial fold; it was asymptomatic and therefore ignored. Within days, two morphologically identical plaques appeared on the adjacent cheek (**Figure 1**). Cosmetic concern prompted referral to our outpatient clinic. Dermoscopy indicated it as a common wart, so he underwent surgical shaving and laser therapy, with specimens submitted for pathological analysis.

**Figure 2:**
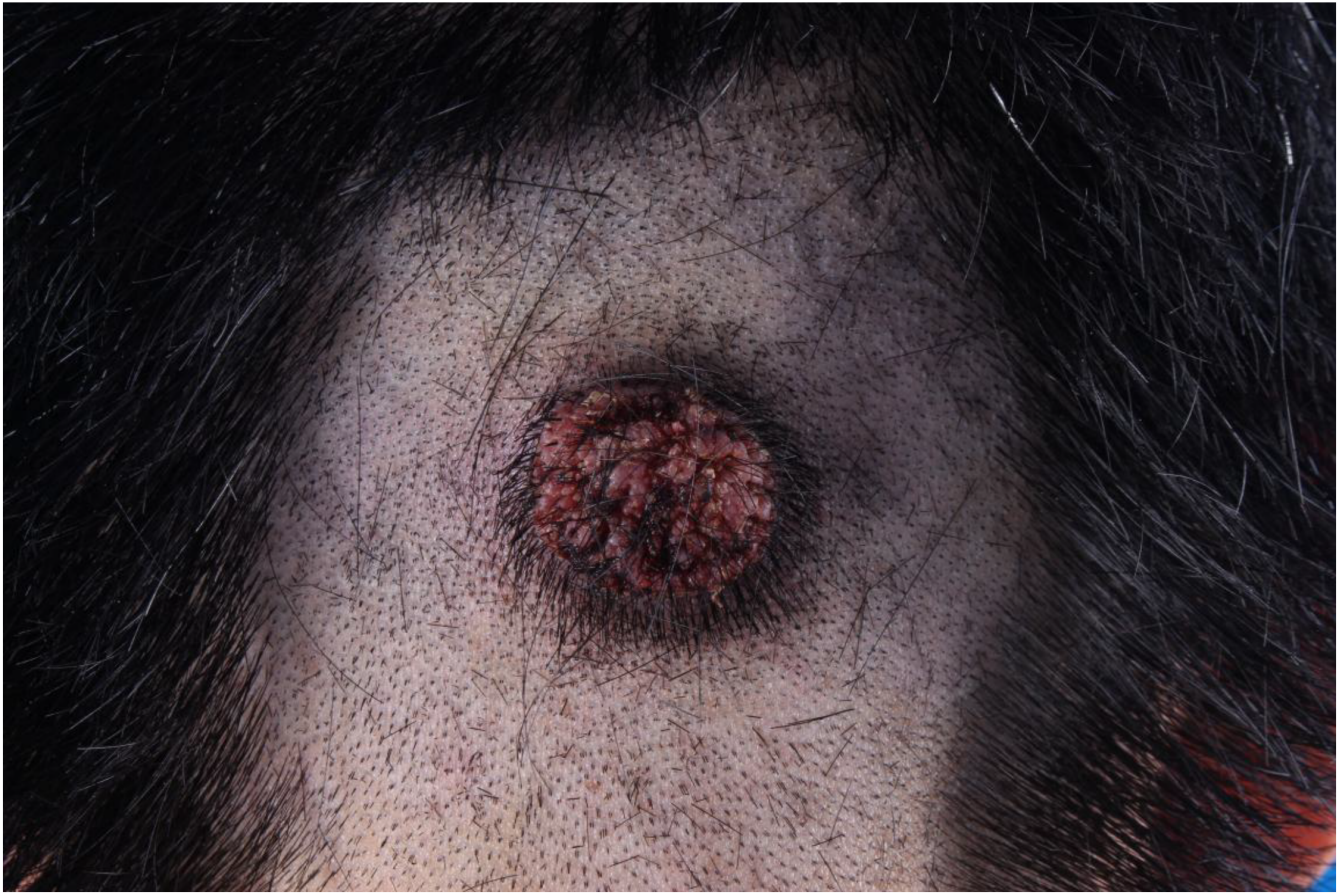
The lesion presents as common wart on the occipital region.

**Figure 3:**
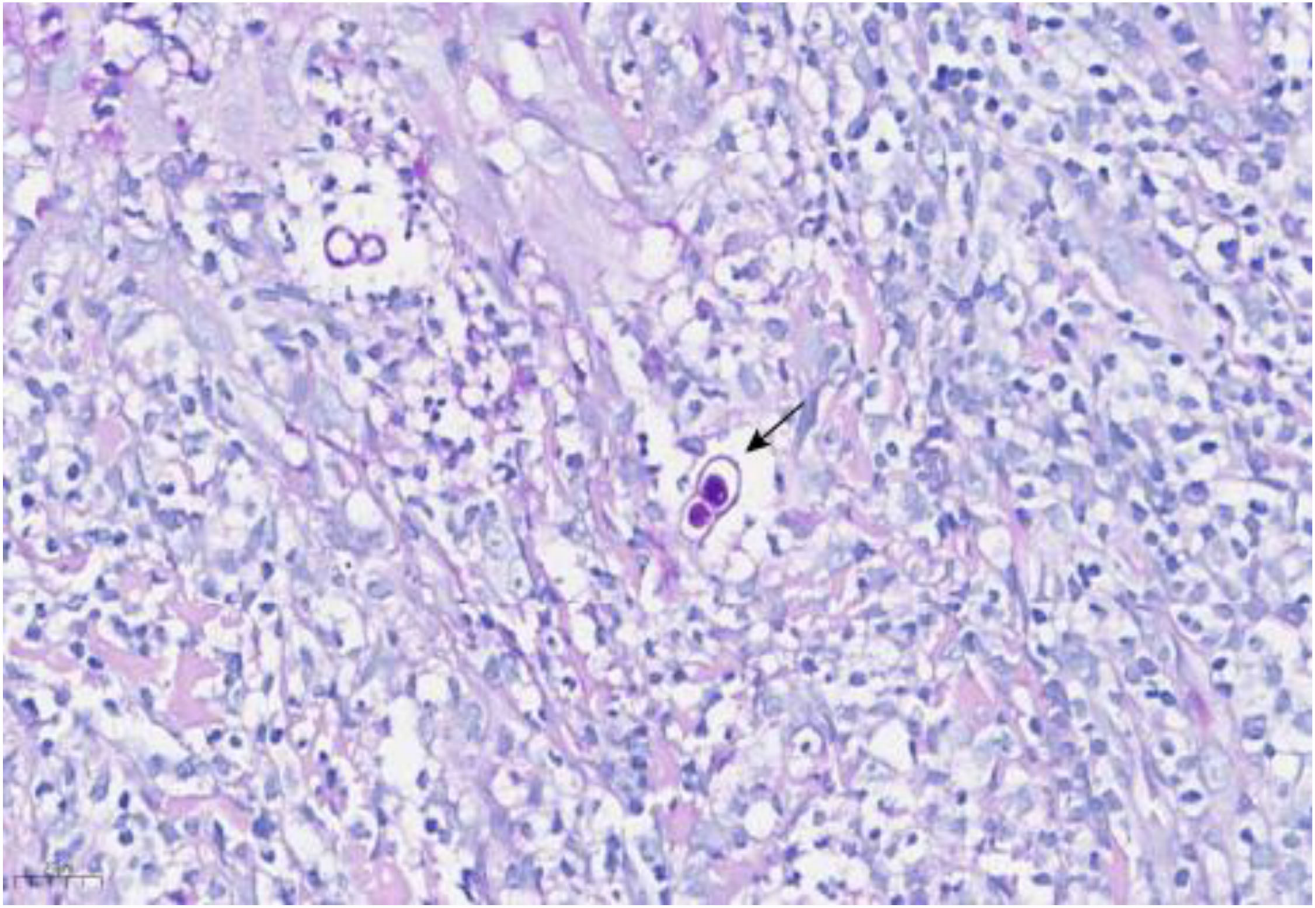
Hematoxylin and Eosin stain (HE 40×) of skin biopsy at the right cheek. Histopathology of skin lesions (Periodic Acid-Schiff staining, 400×). Arrows showed yeast with broad-based budding.

One week after ablation the facial papules recurred; the patient requested repeat laser treatment. Pathological findings revealed infectious granuloma, with negative HPV results. Two weeks later, a similar lesion approximately 0.5-cm verrucous papule appeared on the occipital scalp. Over the next 45 days, the occipital papules expanded and coalesced into a 3.5 cm verrucous plaque (**Figure 2**), while the facial lesions did not recur. He returned for outpatient follow-up, and a biopsy of the occipital lesion confirmed infectious granuloma, with negative acid-fast staining and positive PAS staining (**Figure 3**). Next-generation sequencing (NGS) analysis from the biopsy indicated the presence of *Blastomyces dermatitidis* with a relative abundance of 89.17% and *Histoplasma capsulatum* with a relative abundance of 11.46%.

**Figure 4:**
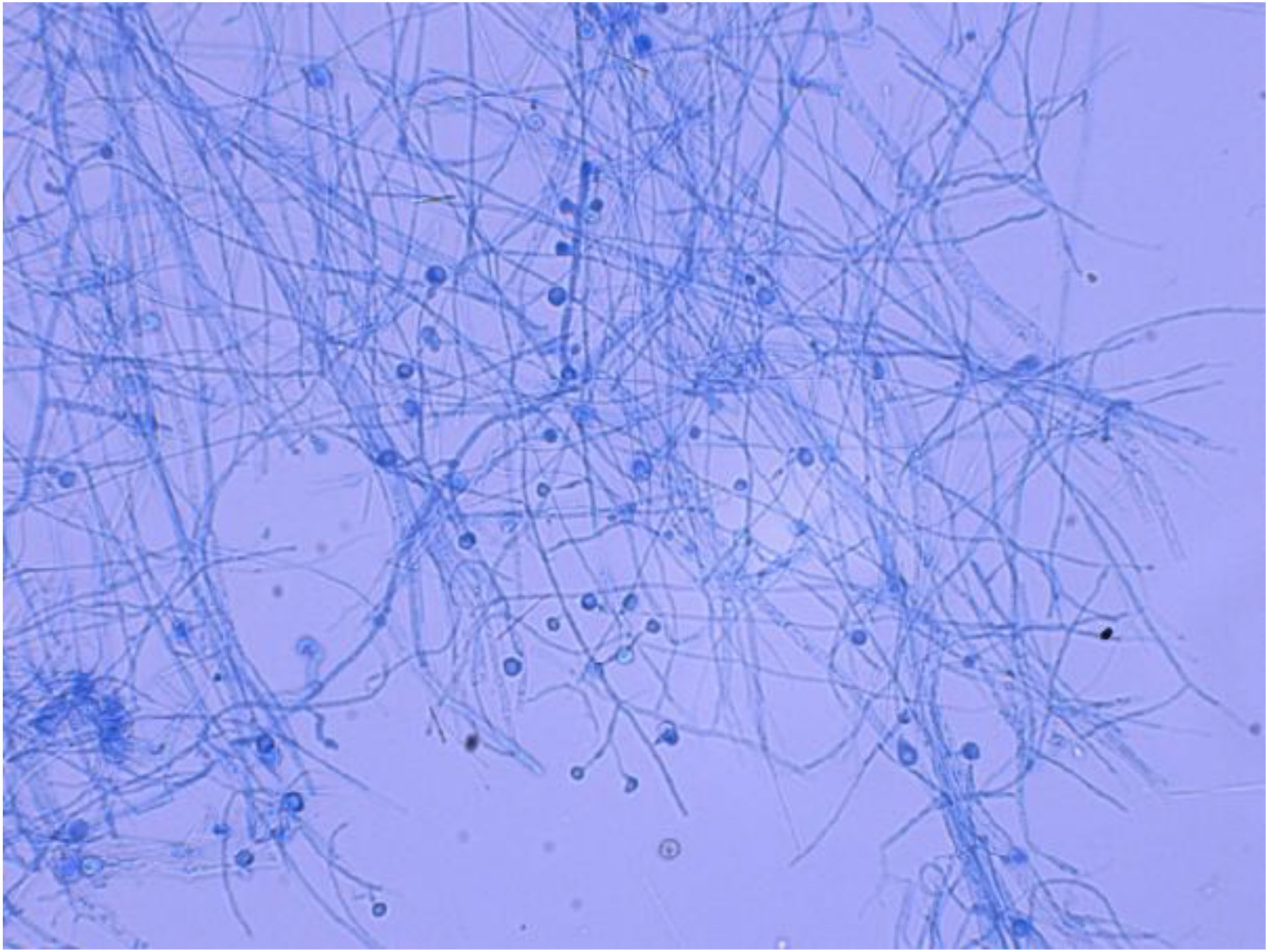
Microscopic examination of the culture on Sabouraud dextrose agar (SDA) medium at 25 °C (gossypol lactate blue staining × 400) showed clear, separated mycelia and apical or lateral pyriform microconidia.

**Figure 5:**
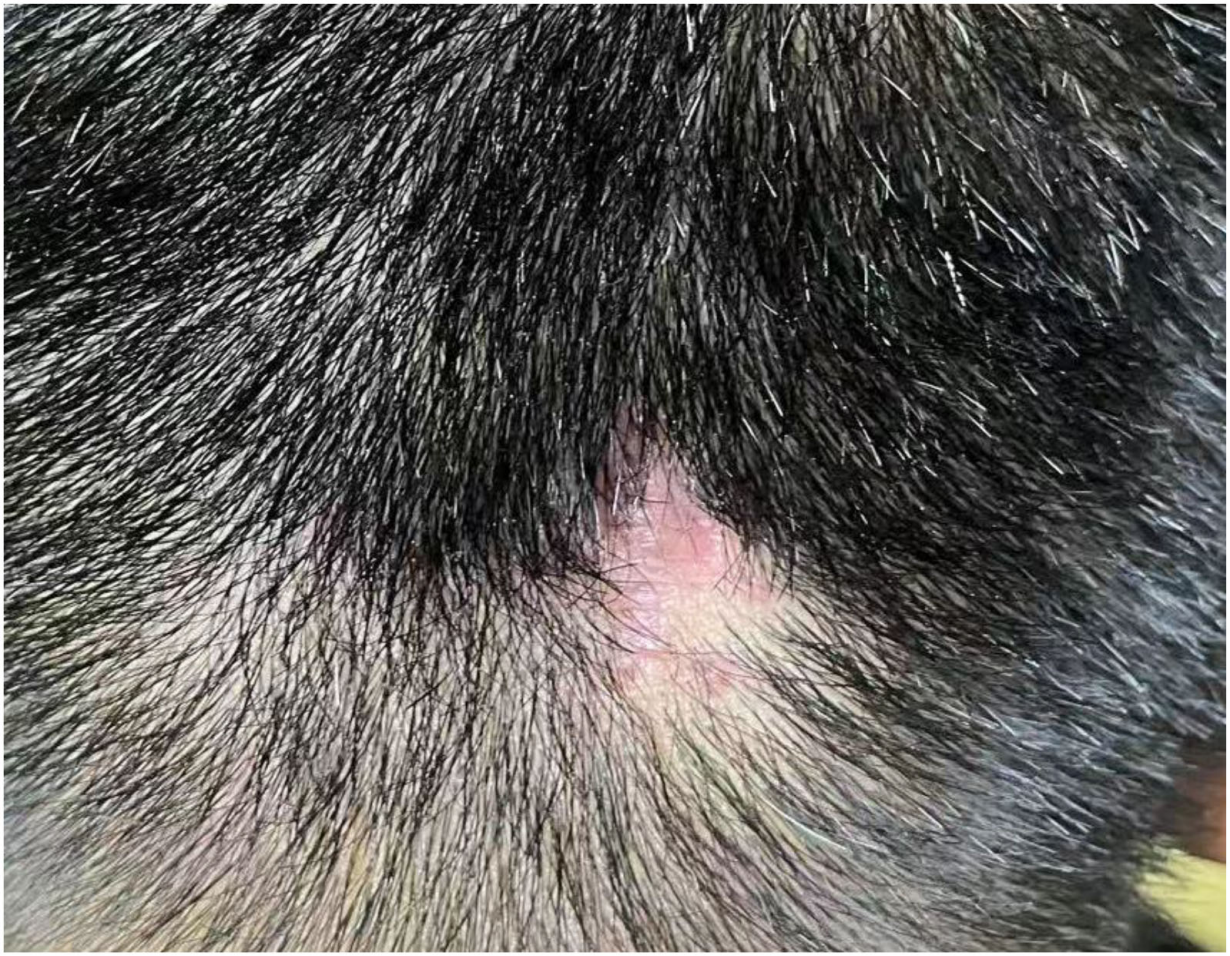
Healing of the lesion after 1 month of itraconazole treatment course.

Pending culture results, the patient was administered itraconazole at a dosage of 0.2 g orally twice daily. To exclude disseminated disease and immunodeficiency, we obtained full blood count, comprehensive metabolic panel, peripheral CD4/CD8 subsets, and serum assays for *G*, *GM*, and *Cryptococcus* test; all were normal. After two weeks, fungal culture revealed the presence of grayish-white waxy colonies. Microscopic examination showed septate hyphae and pear-shaped spores located at the apex or sides (**Figure 4**). The tissue was cut into small pieces to increase surface contact under sterile conditions. The fragments are then aseptically placed onto the surface of Sabouraud dextrose agar (SDA) medium plates. The plates are sealed and incubated at 25 °C and 37 °C for an initial period, respectively. Cultures are examined regularly (every few days) for up to 4 weeks for the appearance of fungal colonies. Any growth is examined macroscopically and microscopically for identification (Huang et al., 2022; Li B. et al., 2025; Li Q. et al., 2025). Molecular sequencing confirmed the isolate as Blastomyces dermatitidis. Further questioning revealed that the patient had lived in Ohio and Indiana for 3.5 years, with only incidental exposure to a Golden Retriever and no recollection of trauma or immunosuppressive therapy. On return to China, CT of the thorax was clear and the patient remained afebrile and asymptomatic from a respiratory standpoint. A diagnosis of primary cutaneous blastomycosis was therefore made. Itraconazole 200 mg twice daily was continued for 6 months. The scalp plaque regressed completely and facial lesions have not recurred (**Figure 5**).

## Discussion

Infections caused by *Blastomyces dermatitidis* predominantly occur in the central-western, southeastern, and south-central regions of the United States, as well as in certain areas of Canada (Canadian provinces that border the Great Lakes, and areas adjacent to the Saint Lawrence Seaway) adjacent to the Mississippi and Ohio River basins (Castillo et al., 2016; Mazi et al., 2021). The majority of infected individuals have a history of environmental exposure, including contact with decaying wood, soil, or activities in moist woodland areas (Linder et al., 2023). The precise prevalence and incidence rates of *Blastomyces dermatitidis* infections remain undetermined. In China, only sporadic cases have been documented over the past three decades, with most cases involving individuals who have traveled to or resided in endemic regions (Zhao et al., 2011). The typical incubation period for *Blastomyces dermatitidis* is generally 4 to 6 weeks (Pullen et al., 2022). Upon reviewing the medical history of our patient, the specific incubation period remains unknown. After residing in China for over one year following his return from an endemic region, we hypothesize that the incubation period of the disease may exceed one year.

*Blastomyces dermatitidis* is capable of infecting nearly every organ in the body, with approximately 25% to 40% of cases resulting in dissemination (Bariola and Vyas, 2011). The most frequently affected sites include the lungs, skin, bones, and genitourinary system (Mazi et al., 2021). Given that the lungs serve as the primary portal of entry for *Blastomyces*, approximately 80% of patients develop pulmonary infections, presenting with symptoms such as cough, fever, night sweats, and fatigue (Kralt et al., 2009).

The skin is the most common site of dissemination in extrapulmonary/disseminated blastomycosis, with cutaneous infections almost invariably secondary to hematogenous spread (Hall et al., 2024). Skin involvement may occur after or concurrently with pulmonary infections in cases of widespread dissemination. In rare instances, isolated cutaneous infections may occur due to direct inoculation, independent of pulmonary involvement (Caldito et al., 2022). Cutaneous lesions typically present on the face and extremities, initially appearing as papules or pustules that progressively develop into verrucous plaques or granulomas, with some lesions potentially evolving into ulcers (Fisher et al., 2009). Prior to accurate diagnosis, these lesions are frequently misidentified as malignant skin tumors (such as basal cell carcinoma or squamous cell carcinoma), bacterial infections (such as tuberculosis), or other dermatological conditions, including common warts, pyoderma gangrenosum, or keratoacanthoma (Rozovsky and Moffatt, 2022).

The clinical presentations, laboratory test results, and radiological findings in patients with *Blastomyces dermatitidis* are often nonspecific. Even in endemic regions, diagnostic delays are common, with more than 40% of cases experiencing a delay of over one month (Mazi et al., 2021). Currently, the diagnosis of *Blastomyces dermatitidis* primarily relies on fungal culture and molecular sequencing for sequence identification, but these methods are time-consuming and not conducive to rapid diagnosis. Metagenomic next-generation sequencing (mNGS) is a technique that enables the unbiased detection of various pathogenic microorganisms through shotgun sequencing of DNA or RNA in clinical samples. This approach allows for the early, rapid, and accurate identification of pathogens in challenging infectious diseases, thereby providing valuable evidence for clinical decision-making.

## Conclusion

We present a case study of a patient infected with blastomycosis dermatitis after traveling to Ohio. Firstly, verrucous plaques occur on the right side of his face before and after laser therapy. However, the results of comprehensive blood tests were normal and the previous CT scan did not reveal any pulmonary lesions. Nevertheless, mNGS analysis indicated the presence of *Blastomyces dermatitidis* and fungal culture revealed the presence of grayish-white waxy colonies. Following treatment with itraconazole for 6 months, the verrucous plaques on the patient’s scalp resolved, and the facial rashes did not recur.

## Data Availability

The raw data supporting the conclusions of this article will be made available by the authors, without undue reservation.
